# Healthfulness of Fruit

**Published:** 1878-05

**Authors:** 


Healthfulness of Fruit.—Fresh, ripe, perfect, raw fruit is safe and healthful at all seasons of the year, and amid the ravages of disease, whether epidemic, endemic or sporadic, general, special or local. Under proper restrictions as to quantity such fruit as named will cure a diarrhœa, aid in removing a cold, colic, fever, or an other disease whose treatment requires the bowels to be kept freely open; for this effect, fresh ripe fruit is acknowledged to have; but to be used advantageously in health and disease the following rules are imperative.

1. Fruit should be eaten ripe, raw, fresh and perfect.
2. It should be eaten in moderation.
3. It should be eaten not later than four o'clock in the afternoon.
4. No water or fluid of any description should be swallowed within an hour after eating fruit.
5. To have its full, beneficial effect, nothing else should be eaten at the time the fruit is taken.

It is to the neglect of these observances that erroneous impressions prevail in many families, and to an extent too, in some instances, that the most luscious peach, or apple, or bunch of grapes is regarded as that much embodied cholera and death. When will men learn to be observant and reflective.

